# The impact of parents’ health behaviours on their preferences regarding vaccinations in Bialystok, Poland

**DOI:** 10.1186/s12887-020-02235-1

**Published:** 2020-07-25

**Authors:** Jolanta Kraśnicka, Elżbieta Krajewska-Kułak, Krystyna Klimaszewska, Mateusz Cybulski, Andrzej Guzowski, Jolanta Lewko, Cecylia Łukaszuk, Krystyna Kowalczuk, Halina Doroszkiewicz, Anna Baranowska, Katarzyna Krajewska-Ferishah, Hanna Rolka, Wojciech Kułak

**Affiliations:** 1 Family Doctors Clinic “Pro Medica Centrum” in Białystok, 15-445 Białystok, Poland; 2 Department of Integrated Medical Care, Medical University of Bialystok, 15-096 Białystok, Poland; 3 Department of Geriatrics, Medical University of Bialystok, 15-471 Białystok, Poland; 4 Department of Pediatric Rehabilitation, Medical University of Bialystok, Waszyngtona 17 street, 15-274 Białystok, Poland

**Keywords:** Vaccinations, Health behaviours, Parents’ opinions

## Abstract

**Background:**

Currently, as the number of vaccinated children in Poland and throughout Europe is decreasing. Many factors impact on the rate vaccination and parents’ health behaviours may affect the frequency of vaccinations. The aim of the study was to assess the association of parents’ health behaviors with children’s vaccinations.

**Methods:**

A cross-sectional survey was conducted from July 2015 to June 2016 to assess to assess the association of parents’ health behaviors with children’s vaccinations in Białystok city, Poland. We used the the Inventory of Health Behaviours and an original questionnaire including demographic data and questions about vaccination. Three hundred parents were recruited from the Pro Medica Family Medica Center in Bialystok, Poland.

**Results:**

Only 3.7% of respondents did not vaccinate their children. The level of health behaviours was average in 42.3% of the respondents, low in 33%, and high in 24.7%. Significant differences in health behaviours, mainly the level of normal eating habits (*p* = 0.038) and positive mental attitude (*p* = 0.022), were found in relation to views on the toxicity of vaccines. Participants who reported that vaccines can cause autism engaged in a higher level of prophylactic behaviours. Respondents who vaccinated their children with combined vaccines had a significantly higher level of health practices.

**Conclusions:**

Parents preferred health behaviours did not effect on children vaccination.

Parents who believed in the toxicity of vaccines were more concerned about proper nutrition, had a positive mental attitude, and engaged in a higher level of preventive behaviours and health practices. Parents who did not vaccinate their children had lower levels of normal eating habits. Parents who vaccinated their children with combined vaccines had a higher level of health behaviours, especially in terms of health practices.

## Background

Many factors can affect childhood vaccination. We believe that parents’ healthy lifestyle behaviors (healthy eating, physical activity, adequate sleep, avoiding stress, non-flammable cigarettes) can affect the frequency of childhood immunizations. It is reasonable to assume that parents who pay attention to their own well-being by making healthy choices are more likely to also monitor their children’s health. We further posit that parents who care more about their health (i.e. exhibit healthy lifestyle behaviors) will be more likely to have their children vaccinated than parents who do not practice healthy lifestyle behaviors. It is well-known that childhood immunizations are important for protection against infectious diseases. In recent months, the Covid-19 pandemic has served to make people aware of the importance of protective vaccinations to limit the risk for infectious diseases that can threaten health and even life.

Health behaviours are a multifactorial set of reactions of social groups associated with treatment and healing agents and stimulated by the need to maintain health and life and prevent disease. Many factors affect both the individual and the environment influence the formation of health behaviours [1–3].

‘Vaccine hesitancy’ is a new term used in last years to describe anyone who is doubtful about vaccinations or who chooses to delay or refuse immunizations even when they are readily available [4]. There are a wide range of factors that cause vaccine hesitancy including the adverse health outcomes, unfamiliarity with vaccine-preventable diseases, lack of trust in public health agencies, and parenteral conspiratorial thinking and needle sensitivity [5, 6]. Reasons for refusing or delaying child vaccination reported by parents differ generally but can be classified into four categories: religious reasons, personal beliefs, safety concerns, and a desire for more information from healthcare providers.

Vaccinations included in the immunisation schedule are mandatory in Poland [7]. This means that every child residing in Poland can receive vaccines funded by the state, as well as that parents are obliged to show up on vaccination visits. At birth, each child receives an immunisation card that is stored at the general practitioner’s (GP) office and used to monitor the immunisation schedule and progress. Based on this card, the GP calls parents for well-baby visits and administers scheduled vaccines as part of developmental monitoring. The current immunization schedule includes 11 mandatory vaccines: tuberculosis, hepatitis B, diphtheria, tetanus, pertussis, poliomyelitis, *Haemophilus influenzae* type b, pneumococci, measles, mumps, and rubella. The immunization schedule also includes a separate section describing which vaccines are recommended (against rotavirus, *Neisseria meningitidis*, hepatitis A, and human *papillomavirus),* but their cost must be paid by the parents.

According to the Polish National Institute of Public Health [8], in Poland in 2017, 98.0% of 2-year-olds, 98.6% of 3-year-olds, 95.5% of 7-year-olds, 93.3% of 11-year-olds, 93.7% of 15-year-olds, 91.7% of 20-year-olds, and 93.3% of girls aged 14 (rubella) received the recommended *vaccines.* Despite the high vaccination rate of children and adolescents in Poland, the number of unvaccinated children has been increasing since 2010. At that time, 0.6% people had avoided their duty to vaccinate, while in 2018, about 5.4% parents refused to vaccinate their children [9]. 2011–2014, the number of people who avoided compulsory immunization more than doubled from approximately 5000 to over 12,000 people [10, 11].

A healthy lifestyle is comprised of the conscious or unconscious choices of a given individual, and vaccination is one of the most important health behaviours. It is worth noting that healthy lifestyle is greatly determined by the social and economic circumstances [12]. Parental vaccination decisions are complex and multi-dimensional.

The family is the first and the most important institution for shaping both attitudes towards maintaining health and the behavioural patterns necessary to maintain health [13]. Parents serve as fundamental role models, although they are not the only examples available to children.

Several studies described the relationship between parents’ health communication behaviors and vaccinations for children [14]. Furthermore, parents with high levels of communicative and critical health literacy are less likely to vaccinate their children [15].

There is a large amount of literature in the immunisation space around parental attitudes [5, 6, 16, 17], but there are no studies assessing the relationship between parental health behavior and vaccination of children. We suggest that parents health behaviours may have impact on vaccination of children.

To the best of our knowledge, there are no studies evaluating the relationship between parental health behaviours and regular immunizations for their children.

The objective of the study was to assess the association of parents’ health behaviors with children’s vaccinations.

## Methods

The study was carried out from July 2015 to June 2016 in Białystok city, Poland. We used the Inventory of Health Behaviour (HBI) [18] which is use for testing different health practices.

The HBI is a Polish questionnaire developed by Juczyński is composed of the statements which describe the behaviors associated with health. It has been used in several Polish studies in obesity patients [19] smokers [20] patients with cancer [21] and elderly people [22]. However, this questionnaire has no international references. The Appendix of the BHI is included. The HBI is intended for the study of healthy and ill adults. It contains 24 statements describing various types of health-related behaviours and enables the determination of both a general indicator of the level of health behaviours and specific indicators for the following categories of behaviours: proper nutrition (mainly taking into account the type of food consumed - including the primary types of food consumed, e.g., whole wheat bread, vegetables, and fruits), preventive behaviours (regarding compliance with health recommendations and obtaining information on health and disease), health practices (daily habits regarding sleep and rest), physical activity (daily exercise habits and recreation), and a positive mental attitude (avoiding strong emotions, stress, depressing situations). The internal reliability of the HBI scale, based on Cronbach’s alpha, is 0.85 for the entire inventory; for the four subscales, internal reliability ranges from 0.60 to 0.65. According to the author’s suggestions regarding the HBI scale, the scores for the health behaviour components were assessed as the average points for each subscale, while the total HBI score was determined by the sum of all obtained points. Thus, eating habits, preventive behaviours, positive mental attitude, and health practices can take values from 1 point (the worst result) up to 5 points (the best result), whereas the total scale score can range from 24 to 120 points, with higher scores indicating greater positive health behaviours.

We also using the diagnostic survey method [23], which is a method of gathering knowledge about structural and functional attributes and the dynamics of social phenomena, opinions, and views of selected communities. The most common techniques used in surveys are interviews, questionnaires, and document analysis. An original questionnaire included demographic data: parents’ age, gender, children’s age, place of residence, education, financial status, and questions about vaccination was using. The vaccination questions were following: vaccination children, immunization is the most effective method of protection against infectious diseases; vaccine safety, compulsory vaccination source of information on vaccination (Details are shown in Tables 2 and 3).

Parents were recruited from the Pro Medica Family Medica Center in Bialystok Poland. It is a family doctors’ outpatient clinic that covered 328 children and the youth population in 2015. The 320 questionnaires were given for parents during doctor visits. The questionnaires were filled by parents in their homes. The completed questionnaires were brought by parents to the clinic. Then the data from the questionnaires were analysed by the authors.

### Data analysis

The data management and analysis were conducted using Statistica version 13.0 (Statsoft, Tulsa, OK, USA). Descriptive analysis was performed by calculating frequencies and percentages of variables. Relationships between two variables were analysed using the chi-square independence test. Relationships among three groups were examined using the Kruskal-Wallis test. The critical level for all tests of significance was *p* < 0.05.

## Results

The 320 questionnaires were delivered, and 314 (98%) were returned. But 300 (94%) surveys were properly completed.

The study included 300 parents; 46.3% were aged 31–40 years, 30% were aged 18 to 30 years, and 23.7% were aged 41 to 50 years. In the studied population, 83% were women, and 17% were men.

A total of 54.3% of the respondents had at least some higher education, 37.3% had a secondary education, 7.3% had a vocational education, and 1% had a primary education. Most had children aged 2 to 4 years (40%), followed by 0–23 months (25.3%), 7–10 years (22.7%), 11–15 years (22.3%), 5–6 years (21%), and 16–18 years (13.3%). Most of the respondents (57.3%) declared that their financial situation was good. The vast majority of the surveyed parents (85%) were not professionally involved in health care. Only 15% of the respondents were in the medical profession.

The vast majority of parents (96.3%) declared that they vaccinated their children. Only 3.7% of the respondents did not vaccinate their children. According to 68% of parents vaccination is the best method of preventing infectious diseases. Similarly 63% of parents reported that vaccines are safe and 65% reported that vaccinations should be compulsory. Most of respondents (84%) said that a family doctor should be a source of information on vaccinations.

In the surveyed population, health behaviours (proper eating habits, preventive behaviours, positive mental attitude and health practices) were practiced at similar levels, although the most frequently occurring health behaviour was a ‘positive mental attitude’. Details are shown in Table 1.

**Table 1:** Parents health behaviours assessed using the HBI scale

HBI	$\overline{x}$	Me	*s*	*Q*_25_	*Q*_75_	min	max
Proper eating habits	3.4	3.5	0.7	3.0	3.8	1.5	5.0
Preventive behaviours	3.5	3.5	0.7	3.0	4.0	1.5	5.0
Positive mental attitude	3.6	3.7	0.6	3.2	4.0	1.5	5.0
Health practices	3.2	3.2	0.6	2.8	3.7	1.3	4.7
Parents level of health behaviours	Number of parents			**%**			
Low	99			33.0%			
Average	127			42.3%			
High	74			24.7%			

*HBI* Health Behaviour Inventory $\overline{x}$ - mean, *Me* Median, *s* Standard error, *Q* Quartile

Five-point scale of the HBI: 1- almost never, 2- rarely, 3- occasionally, 4- often, 5- almost always

The total HBI scale rates health behaviours on three levels: low, average, and high. Almost half of the parents (42.3%) had an average level of health behaviours; one in three respondents had a low level, and one in four had a high level. Details are provided in Table 1.

Next, the relationships among behaviour levels in the four areas specified on the HBI questionnaire was examined. The level of individual health behaviours was measured on a numerical scale. The analysis consisted of juxtaposing the mean HBI scores (with standard deviations) by group with the responses to the questions regarding vaccination and determining the significance of the relationship between them using the Kruskal-Wallis test. Opinions on immunity being infected with a disease were not related to the parents’ health behaviours. Opinions on the need to vaccinate for all diseases, the vaccination system, and vaccine quality were not related to health behaviours. Details are shown in Table 2.

**Table 2:** Selected opinions of parents depending on their preferred health behaviours

	Proper eating habits		Preventive behaviour		Positive mental attitude		Health practices	
	$\overline{x}$	*s*	$\overline{x}$	*s*	$\overline{x}$	*s*	$\overline{x}$	*s*
It is better to develop resistance by being infected than by vaccination								
**yes**	3.47	0.64	3.57	0.67	3.78	0.61	3.45	0.61
**no**	3.42	0.64	3.54	0.66	3.58	0.60	3.20	0.61
**hard to say**	3.33	0.75	3.37	0.77	3.52	0.67	3.15	0.63
***p***	0.5632		0.2366		0.2531		0.1096	
You should be vaccinated against all diseases								
**yes**	3.38	0.69	3.50	0.66	3.57	0.64	3.10	0.61
**no**	3.42	0.69	3.45	0.69	3.58	0.60	3.29	0.58
**hard to say**	3.40	0.63	3.56	0.70	3.58	0.62	3.21	0.64
***p***	0.9595		0.2819		0.8752		0.1326	
The current vaccination programme takes into account the situation in Poland								
**yes**	3.40	0.67	3.50	0.75	3.55	0.66	3.19	0.64
**no**	3.49	0.61	3.59	0.61	3.58	0.49	3.18	0.58
**hard to say**	3.36	0.68	3.46	0.59	3.62	0.59	3.24	0.59
***p***	0.7630		0.6118		0.8036		0.7126	
The vaccines used in Poland are safe								
**yes**	3.40	0.64	3.52	0.71	3.58	0.62	3.19	0.63
**no**	3.60	0.67	3.58	0.56	3.71	0.56	3.00	0.59
**hard to say**	3.37	0.72	3.46	0.66	3.56	0.63	3.27	0.58
***p***	0.8341		0.7924		0.8125		0.3811	
Vaccines contain toxic ingredients								
**yes**	3.67	0.66	3.67	0.55	3.80	0.52	3.36	0.61
**no**	3.40	0.68	3.49	0.73	3.61	0.64	3.22	0.65
**hard to say**	3.32	0.64	3.46	0.68	3.49	0.61	3.16	0.58
***p***	0.0381*		0.3287		0.0223*		0.1416	
Vaccines cause developmental disorders and autism								
**yes**	3.63	0.56	3.80	0.61	3.90	0.64	3.42	0.62
**no**	3.39	0.71	3.41	0.73	3.57	0.65	3.18	0.63
**hard to say**	3.37	0.63	3.54	0.64	3.54	0.57	3.20	0.59
***p***	0.2963		0.0375*		0.0773		0.2425	
Vaccinations should be mandatory								
**yes**	3.43	0.63	3.50	0.71	3.55	0.63	3.20	0.63
**no**	3.32	0.68	3.53	0.66	3.71	0.58	3.27	0.56
**hard to say**	3.33	0.82	3.37	0.58	3.38	0.62	3.08	0.66
**only some**	3.62	0.77	3.67	0.51	3.60	0.44	3.21	0.37
***p***	0.5949		0.5182		0.1777		0.8082	
Children who are not vaccinated should not be admitted to kindergartens and crèches								
**yes**	3.40	0.64	3.48	0.70	3.52	0.67	3.16	0.68
**no**	3.43	0.68	3.62	0.63	3.60	0.57	3.21	0.63
**hard to say**	3.37	0.69	3.42	0.71	3.63	0.59	3.27	0.51
***p***	0.8455		0.3155		0.5410		0.5826	
I have doubts about vaccinating my child								
**yes**	3.40	0.77	3.52	0.65	3.63	0.55	3.28	0.58
**no**	3.40	0.61	3.55	0.66	3.62	0.62	3.19	0.61
**hard to say**	3.38	0.68	3.26	0.82	3.30	0.67	3.15	0.70
***p***	0.9613		0.0823		0.0156*		0.4125	

$\overline{x}$ Mean, *s* Standard deviation; Five-point scale of the HBI: 1- almost never, 2- rarely, 3- occasionally, 4- often, 5- almost always

Significant differences in health behaviour levels, particularly eating habits (*p* = 0.038) and positive mental attitude (*p* = 0.022), were found among respondents with different opinions on the toxicity of vaccines. Parents who believed that vaccines were toxic cared about proper nutrition had positive mental attitudes and a higher level of preventive behaviours and health practices than other parents. Respondents who believed that vaccines can cause autism had a significantly higher level of preventive behaviours than other parents. Opinions on the general obligation to vaccinate were not related to health behaviour levels in any of the four areas identified in the HBI questionnaire. Additionally, opinions on the admission of unvaccinated children to crèches and kindergartens did not relate to the respondents’ health habits. Details are shown in Table 2.

Parents who did not vaccinate their children with combined vaccines had a lower level of normal eating habits (*p* = 0.058). Parents who vaccinated their children with combined vaccines showed significantly higher levels of health practices (on average, 3.28 for this group compared with 3.12–3.13 in the other two groups). Significant or near-significant differences in levels of various health behaviour were found between those who used recommended vaccinations and those who did not. Parents who vaccinated their children also had a higher level of health behaviours, especially in the field of health practices. Details are shown in Table 3.

**Table 3:** Preference for child vaccination according to parents’ preferred health behaviours

	proper eating habits		preventive behaviour		positive mental attitude		health practices	
	$\overline{x}$	*s*	$\overline{x}$	*s*	$\overline{x}$	*s*	$\overline{x}$	*s*
Has your child been vaccinated with combined vaccines?								
**yes**	3.47	0.60	3.57	0.64	3.63	0.59	3.28	0.61
**no**	3.29	0.71	3.42	0.73	3.52	0.66	3.12	0.62
I do not remember	3.58	0.88	3.43	0.85	3.50	0.48	3.13	0.54
***p***	0.0584		0.2043		0.1620		0.0245*	
Has your child had the recommended vaccinations?								
	$\overline{x}$	*s*	$\overline{x}$	*s*	$\overline{x}$	*s*	$\overline{x}$	*s*
**yes**	3.47	0.65	3.59	0.69	3.62	0.67	3.28	0.66
**no**	3.31	0.62	3.40	0.68	3.56	0.56	3.12	0.57
I do not remember.	3.38	0.95	3.39	0.63	3.36	0.52	3.14	0.48
***p***	0.1009		0.0783		0.0868		0.0380*	

$\overline{x}$ Mean, *s* Standard deviation; Five-point scale of the HBI: 1- almost never, 2- rarely, 3- occasionally, 4- often, 5- almost always

## Discussion

In the present study, parents preferred health behaviours did not effect on children vaccination. Only 3.7% of respondents did not vaccinate their children. Furthermore, parents with preferred health behaviours (proper eating habits and positive mental attitude) significantly more often reported that vaccines contain toxic ingredients. Also, parents with preventive behaviours more often reported that vaccines cause developmental disorders and autism. And, parents with positive mental attitudes significantly more often had doubts about vaccinating their children. Parents who vaccinated their children with combined and recommended vaccines showed significantly higher levels of health practices.

The present findings suggest less importance of parenteral health behaviour on children vaccination. It well known [24, 25] that many factors impact on immunisation rates for example: social determinants such as young age of parents, level of parental education, family income, lack of health insurance, lack of periodic primary health care access, or pay for vaccines.

The role of the parents’ perceptions health beliefs and attitudes toward childhood immunization [26] are risk factors for decreased vaccination. Other studies [27–30] suggest that socioeconomic factors play a more important role, and parents’ beliefs may simply reflect their sociodemographic characteristics.

Our results on rate vaccination are consistent with a recent study from the United States [30]. Ninety-six percent of parents reported that their children had received all vaccines recommended for children up to their age. Moreover, 3.5% of all parents indicated they had decided not to have their child/children get a recommended vaccine. As in our study, some parents reported that vaccines have ingredients that are unsafe (35%) and 19% believed that vaccines may cause as autism.

Also, similar rate children immunisation was found in a study from Israel [31] where 90–89% children had full immunisation in the years 2008 and 2016. However, a declining confidence of parents in official recommendations for vaccination from 87 to 72% was demonstrated.

It is believed that greater health behaviour awareness in the family results in better the health effects for its individual members [32]. The present and future health of the family system and all its members depends largely on the parents’ actions, decisions, conduct, choices, and preferred lifestyle. It should be noted that parents raise children based not only on scientific and popular science knowledge but also on colloquial knowledge. These types of knowledge may have a positive or negative influence on the development of family behaviour patterns, the introduction of modifications and changes in behaviours and the consolidation of previously understood attitudes, including those related to health [10].

In the currently studied population, all categories of studied health behaviours (proper eating habits, preventive behaviours, a positive mental attitude, and health practices) were at a similar level; however, the most frequent behaviours was a positive mental attitude.

In the literature on the subject, health behaviours include the conscious undertaking of health-oriented actions (various behaviours related to physical health, mental health, self-management of health, preventive examinations, safe behaviours in everyday life, common sense behaviour regarding diseases) and the elimination of all activities that pose a threat to life and health, both directly and in the long term (e.g., abstaining from tobacco, alcohol, recreational drugs, and psychoactive substances) [1, 3]. One of the most important health behaviours is vaccination, which is the most effective preventive method for combating diseases.

A survey of a representative sample of 990 adults in Poland showed that 79% of respondents considered vaccinations the most effective way to protect children from serious diseases. The vast majority of the surveyed parents (96.3%) declared that they vaccinated their children. The aforementioned survey of a representative random sample of 990 adults in Poland showed that 79% of respondents thought that vaccinating children causes more good than harm [11].

The present study showed that parents’ health behaviours were not statistically correlated their beliefs regarding the vaccination system, the quality of the vaccines used in Poland, the general obligation to gain immunity through “sickness,” the need to vaccinate against all diseases and the acceptance of unvaccinated children in nurseries and kindergartens.

In a recent study from 2020, including 5736 parents from 18 country European, survey on parents’ attitudes and behaviours regarding their children’s immunization was performed. Fifty six percent respondents described themselves as “not at all hesitant”, and 24% respondents “somewhat hesitant”, respectively. Vaccine confidence was highest in Portugal and Cyprus, and lowest in Bulgaria and Poland [32].

At present, more and more parents avoid vaccinating their children. Negative opinions that undermine the effectiveness and safety of preventive vaccinations as a form of infection prevention are widely disseminated [4, 5]. There are so-called anti-vaccine movements that aim to reduce mass vaccination. On online portals and forums, there is information regarding the harmfulness of preventive vaccinations, complications arising from vaccinations, and the impact of mercury on the emergence of autism, autoimmune diseases, or weakening of the body’s resistance. Anti-vaccine content is also spread among those who use homeopathic and natural medicine.

In the present study, parents who believed that vaccines can cause autism showed a significantly higher level of prophylactic behaviour. Statistically, there were significant differences in health behaviour levels related to views on the toxicity of vaccines, mainly in terms of proper eating habits and a positive mental attitude. Furthermore, 8% of the respondents reported that vaccines cause autism, 45% had no opinion, and 47% reported that vaccines do not cause autism. But only 3.7% did not vaccine their children. Parents beliefs that vaccines can cause autism are complex [30]. However parents’ belief that vaccination causes autism, twice as many parents vaccinated their children. Which may suggest that parents are not always telling the truth when completing the survey.

And our beliefs come from our values. A negative example of the dissemination of false information/ beliefs regarding vaccinations was the investigation by Wakefield, who put forward the concept of a relationship between the Measles, Mumps and Rubella Vaccine (MMR) vaccination and autism [33].

The most common reason for hesitancy or refusal for MMR is autism which was conformed in many previous studies [34, 35]. Furthermore, vaccine-hesitant parents in Switzerland believed the risks of vaccination were worse than measles itself [36].

It is worth emphasizing the limitations of this study. First, this study involved relatively small group of parents, mainly woman. Secondly, parents were of different ages and had different educational level. Finally, 15% of the respondents were in the medical professions.

In summary, it is worth re-emphasizing that the family, as the most important, most basic social group on which society is based, should provide future generations with educational development, including vaccinations, based on cooperation with health care units and other educational units.

## Conclusions

In the present study, parents preferred health behaviours did not effect on children vaccination. Parents who believed in the toxicity of vaccines were more concerned about proper nutrition, had a more positive mental attitude, and had a higher level of preventive behaviours and health practices. Parents who did not vaccinate their children with combined vaccines had lower levels of normal eating habits, and those who vaccinated their children had a higher level of health behaviours, especially in the area of health practices.

## Data Availability

The data analysed during the current study are available from the corresponding author on reasonable request.

## Appendix

### The health behaviour inventory

The Health Behaviour Inventory (HBI*)* by Juczynski *(translated from Polish to English).*

Please put the X in place of ▢ next to one of the selected answers.

**Table:**

		**Almost never**	**Rare**	**From time to time**	**Often**	**Almost always**
**1**	I eat a lot of vegetables and fruits					
**2**	I avoid colds					
**3**	I take seriously the tips of people expressing concern about my health					
**4**	I rest enough					
**5**	I limit the consumption of products such as animal fats and sugar					
**6**	I have phone numbers of the emergency services					
**7**	I avoid situations that depress me					
**8**	I avoid overworking					
**9**	I care about proper nutrition					
**10**	I follow medical recommendations resulting from my tests					
**11**	I try to avoid too strong emotions, stresses and tensions					
**12**	I control my weight					
**13**	I avoid eating food with preservatives					
**14**	I regularly report for medical examinations					
**15**	I have friends and a regulated family life					
**16**	I sleep enough					
**17**	I avoid salt and highly salted food					
**18**	I’m trying to find out how others avoid disease					
**19**	I avoid feelings such as anger, anxiety and depression					
**20**	I limit smoking					
**21**	I eat whole wheat bread					
**22**	I am trying to obtain medical information and understand the causes of health and illness					
**23**	I think positive					
**24**	I avoid excessive physical activity					

#### The health behaviour inventory (HBI) by Juczynski

It is intended for examining healthy and sick adults. It contains 24 statements describing various types of health-related behaviors (eating habits, preventive behaviors, positive mental attitudes, health practices). It allows to set a general indicator of the severity of health behaviors and the severity of four categories of these behaviors: proper nutrition (mainly taking into account the type of food consumed), preventive behavior (regarding compliance with health recommendations and obtaining information on health and illness), health practices (daily sleep habits and rest and physical activity), a positive mental attitude (avoiding strong emotions, stress, depressing situations).

The internal compliance of HBI, determined on the basis of Cronbach's alpha, is 0.85 for the entire Inventory, while for its four subscales it ranges from 0.60 to 0.65. In a test-retest study, conducted on 30 people, six weeks apart, a correlation index of 0.88 was obtained.

Taking into account the frequency of individual behaviors indicated by the respondents, the overall severity of health-promoting behaviors and severity of four categories of health behaviors, i.e. correct eating habits, preventive behaviors, health practices and a positive mental attitude are determined.

Correct eating habits primarily take into account the type of food consumed (e.g. whole grain bread, vegetables and fruit). Preventive behaviors relate to compliance with health recommendations, obtaining information on health and illness. Health practices include daily sleep and recreation or physical activity habits. Positive mental attitudes include in the scope of health behaviors such psychological factors as avoiding too strong emotions, stress and tension, or situations depressingly.

The respondent indicated how often he performed the given health-related activities, assessing each of the behaviors listed in the inventory on a five-point scale: 1- almost never, 2- rarely, 3- occasionally, 4- often, 5- almost always.

Due to the possibility of periodic preference for certain types of health behaviors, it was assumed that the assessment should take into account the last year. Numerical values marked by the subject were counted in order to obtain within 24 to 120 points. The higher the result obtained by the respondent, the greater was the severity of his health behaviors.

The general indicator, after transformation into standardized units based on the table below, was interpreted according to the properties characterizing the sten scale. Results within:

- 1–4 sten were assumed to be low
- 7–10 sten - as high,
- 5 and 6 sten - as average.

**Polish norms HBI**

**Table:**

Men ***N*** = 235	Sten	Women ***N*** = 261
Raw result		Raw result
24–50	1	24–53
51–58	2	54–62
59–65	3	63–70
66–71	4	71–77
72–78	5	78–84
79–86	6	85–91
87–93	7	92–98
94–101	8	99–104
102–108	9	105–111
109–120	10	112–120

In addition, the severity of four categories of health behaviors is calculated separately - the indicator was the average number of points in each category, i.e. the sum of points divided by 6. The diagnostic key is given in the next table.

**HBI diagnostic key**

**Table:**

Behavior categories	Item numbers
Correct eating habits	1,5, 9,13,17,21
Preventive behavior	2, 6, 10, 14, 18, 22
Positive mental attitudes	3, 7, 11, 15, 19, 23
Health practices	4, 8, 12, 16, 20, 24

Juczyński Z: Narzędzia pomiaru w promocji i psychologii zdrowia. Wyd. Pracownia Testów Psychologicznych, Warszawa 2009

## Abbreviation

HBI: Health Behavior Inventory
